# Cancer Stem Cells in Head and Neck Squamous Cell Carcinoma

**DOI:** 10.1155/2011/762780

**Published:** 2010-11-08

**Authors:** Marcus M. Monroe, Eric C. Anderson, Daniel R. Clayburgh, Melissa H. Wong

**Affiliations:** ^1^Department of Otolaryngology—Head and Neck Surgery, Oregon Health & Science University, 3181 SW Sam Jackson Park Rd, Portland, OR 97239, USA; ^2^Division of Hematology and Medical Oncology, Oregon Health & Science University, 3181 SW Sam Jackson Park Rd, Portland, OR 97239, USA; ^3^Department of Dermatology, Cell and Developmental Biology, Knight Cancer Institute, Oregon Stem Cell Center, Oregon Health & Science University, 3181 SW Sam Jackson Park Rd, Portland, OR 97239, USA

## Abstract

Accumulating evidence suggests that self-renewal and differentiation capabilities reside only in a subpopulation of tumor cells, termed cancer stem cells (CSCs), whereas the remaining tumor cell population lacks the ability to initiate tumor development or support continued tumor growth. In head and neck squamous cell carcinoma (HNSCC), as with other malignancies, cancer stem cells have been increasingly shown to have an integral role in tumor initiation, disease progression, metastasis and treatment resistance. In this paper we summarize the current knowledge of the role of CSCs in HNSCC and discuss the therapeutic implications and future directions of this field.

## 1. Introduction

Head and neck squamous cell carcinoma (HNSCC) ranks sixth worldwide for cancer-related mortality, with an estimated 500 000 new cases diagnosed yearly [1]. For the past several decades the mainstay of treatment for HNSCC has been surgery and external beam radiation. More recent clinical trials have demonstrated the benefit of combining chemotherapy and radiation for advanced stage disease, leading to modest improvements in treatment outcomes [1–5]. Despite this recent improvement, the increase in overall survival has been nominal and cancer recurrence and treatment failures continue to occur in a significant percentage of patients. The biology underpinning why some tumors respond favorably to treatment and others do not is largely unknown. 

Over the last 15 years, advances in tumor biology have led to the discovery that many cancers, including HNSCC, appear to be supported by cells with stem-like properties. Studies in a wide variety of malignancies have demonstrated that only a distinct subpopulation of tumor cells, termed cancer stem cells (CSCs), contain the ability to undergo self-renewal and differentiation (properties of normal stem cells) and hence have the ability to initiate tumorigenesis and support ongoing tumor growth. Furthermore, it appears that, like their normal stem cell counterparts, CSCs have increased resistance to standard cytotoxic therapies. These findings have coalesced into the cancer stem cell theory of tumorigenesis, which has remarkable implications on our understanding of tumor initiation, disease progression, and treatment response. Here we review the basics of the cancer stem cell theory as it applies to HNSCC.

## 2. Emergence of the Cancer Stem Cell Theory

**Figure 1:**
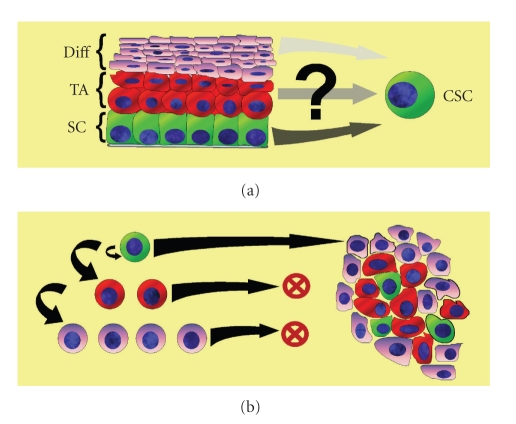
CSC Theory. (a) Origin of CSC. CSC may originate from endogenous stem cells (SC) or reprogramming of the transit amplifying (TA) or differentiated (Diff) cell population. (b) The CSC theory proposes a tumor cell hierarchy with the CSC at the apex. Only CSCs are able to give rise to new tumors and provide support for ongoing tumor growth (Green: CSC, Red: Transit amplifying population, Pink: differentiated cell population).

The cellular and molecular requirements for initiation of tumorigenesis are a series of mutations resulting in the acquisition of replication and growth-factor independence, resistance to growth-inhibitory signals, tissue invasion, and metastasis [6]. The mechanisms underlying these mutations have been extensively interrogated; however a unifying “model of tumorigenesis” remains to be completely elucidated. Tumors have long been recognized to consist of a heterogeneous population of cells differing in proliferative capacity, histologic and immunophenotypic appearance, and tumorigenic potential. Traditionally, this heterogeneity has been hypothesized to be the result of the stochastic accumulation of numerous and varied individual mutations and microenvironmental signals that provide a selective advantage to certain tumor cells. Over the last several years however, a new hypothesis has emerged suggesting that tumor heterogeneity is supported by a stem cell hierarchy. The cancer stem cell hypothesis postulates that tumor heterogeneity with regards to initiation, progression, response to therapy, and metastasis is the result of mutations which either render a normal somatic tissue stem cell cancerous, or cause a cancer cell to become stem cell-like [7]. This mutated CSC is then capable of giving rise both to additional CSCs and to a variety of more differentiated and functionally divergent cancer cells, much like a normal somatic tissue stem cell. Unlike in the traditional stochastic tumorigenesis model, the CSC model proposes that tumorigenicity resides in only a small subpopulation of cancer cells and that these cells, rather than the bulk of the tumor, are responsible for tumor initiation and growth (Figure 1).

As with normal somatic stem cells, CSCs are defined by their ability to self-renew and to give rise to a heterogeneous population of tumor cells. This population of tumor cells consists of rapidly dividing cells (similar to the transient amplifying (TA) cell population in normal tissue) as well as additional CSCs and more differentiated tumor cells. In addition to their replicative capacity, CSCs, like their somatic counterparts, are also more resistant to the effects of cytotoxic chemotherapies and radiation damage [8–16]. Defining this stem cell hierarchy and the complex relationship between these cell populations has critical implications, not only for the understanding of the biology of tumor initiation and progression, but also for prognosis and treatment.

CSCs were first experimentally defined in hematopoietic malignancies by John Dick and colleagues in 1994 [17]. Transplantation of a defined subpopulation of human acute myeloid leukemia (AML) cells (CD34^hi^ CD38^low^) into immunodeficient mice was not only able to recapitulate AML but it was phenotypically and pathologically similar to the patient's original leukemia. In contrast, the remaining cell populations (CD34^low^ and CD34^hi^ CD38^hi^) failed to give rise to new leukemia cells.

In the 15 years since the identification of the leukemic stem cell, a number of investigators have identified CSCs in solid malignancies. In 2003, Michael Clarke and colleagues were the first to identify a CSC population in a solid tumor. A subpopulation of CD44^hi^ CD24^low^ breast cancer cells were able to recapitulate phenotypically heterogeneous breast cancers at very low limiting dilutions in mouse xenograft experiments [18]. Since then a number of other groups have defined CSC populations in other epithelial malignancies including colorectal, prostate, lung, brain, and HNSCC [19–23]. The identification of the cell population responsible for initiating tumorigenesis has significant implications for the prognosis and treatment of cancer. At present, cytotoxic chemotherapies target the rapidly cycling cells of the tumor and result in impressive reduction in tumor size, but leave the largely chemotherapy-resistant CSCs untouched [10, 15, 24–26]. Additionally, both *in vitro* assays and *in vivo* monitoring for effectiveness of new experimental cancer therapies are based on reduction in cell number or tumor size. It is therefore theoretically possible that therapies which result in tumor cell death, as currently assayed, will not have any significant effect on the CSC and will therefore not result in long-term disease control or eradication. The ability of the CSC to produce phenotypically diverse tumor cells may also contribute to increased metastatic potential with new mutations selecting for migratory and invasive properties of the tumor.

## 3. Endogenous Head and Neck Stem Cells and the Origin of Cancer Stem Cells

The origin of the cancer-initiating cell has long been presumed to be the normal endogenous tissue stem cell. This is based upon their similar behaviors and the notion that only accumulated mutations within a long-lived cell could ultimately result in tumorigenesis. In colorectal cancer there is a strong correlation between induced loss of the Wnt signaling molecule APC in a putative stem cell population and the formation of benign intestinal polyps [27], providing evidence that intestinal cancers can arise from a progenitor population. However, it is possible that accumulation of genetic mutations within a differentiated or progenitor cell can allow expression of stem cell behavior, and that this may provide an alternative source of CSCs. For example, oncogene expression driven from myeloid-specific promoters resulted in generation of mouse models of human leukemias [28, 29]. Despite focused examination, the origin of the CSC remains controversial. 

With the primary focus on identifying CSC markers in HNSCC, little is known about the identity or the location of the normal endogenous stem cell or the stem cell microenvironment. Several studies have examined the putative HNSCC CSC marker CD44 in normal head and neck epithelia with differing conclusions. In one study, isolated CD44^hi^ normal oral keratinocytes were shown to exhibit a G2-block associated with apoptosis resistance, a potential stem cell feature [30], suggesting that CD44 is likely expressed in normal head and neck epithelial stem cells. However, a subsequent study demonstrated that 60%–95% of the normal epithelia express CD44 (or 60%–80% the splice variant CD44 v6), far too many cells to be considered tissue stem cells. While CD44 populations may indeed harbor a subpopulation encompassing stem cells, by itself it does not appear to be an adequate stem cell marker for normal oral mucosa [31]. The head and neck stem cell identity and niche is clearly underexplored, however, key insights from the skin [32, 33], airway mucosa [34] and esophagus [35] may guide future investigations into elucidation of this stem cell population.

## 4. Cancer Stem Cell Identification

**Figure 2:**
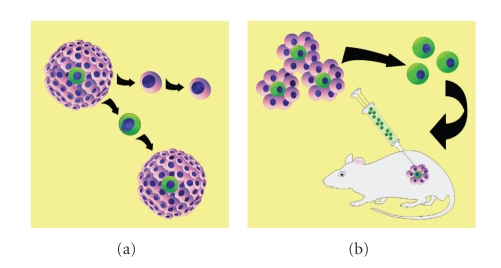
CSC Identification. (a) Tumor sphere formation by CSCs. Differentiated tumor cells (pink) are unable to give rise to new clonally derived tumor spheres, whereas CSCs (green) give rise to new tumor spheres, (b) Tumor subpopulations are identified through differing mechanisms, including cell surface markers, aldehyde dehydrogenase activity, side population and are isolated with fluorescence-activated cell sorting (FACS). Xenotransplantation assays in immunocompromised mice demonstrate tumorigenic, self-renewing and differentiation properties of putative CSC population.

Methods for the identification of CSCs in solid malignancies mirror those strategies employed to differentiate normal stem cells from their differentiated progeny. These include the efflux of vital dyes by multidrug transporters, the enzymatic activity of aldehyde dehydrogenase, colony and sphere-forming assays utilizing specific culture conditions and the most widely used method—the expression of specific cell surface antigens known to enrich for stem cells. Once the subpopulation of tumor cells has been identified and isolated, functional characterization through quantitative xenotransplantation assays, the gold-standard for identification of CSCs, are used to assess the tumorigenicity and self- renewing potential of the putative CSC population *in vivo* (Figure 2).

### 4.1. Surface Antigens

 By far the most common method of identifying CSCs has relied on the expression of specific cell-surface antigens that enrich for cells with CSC properties. Many of these antigens were initially targeted because of their known expression on endogenous stem cells. While a multitude of studies have identified CSC markers across a variety of solid malignancies, relatively few of these markers have been studied in HNSCC.

#### 4.1.1. CD133

A pentaspan transmembrane glycoprotein localized on cell membrane protrusions [36, 37], is a putative CSC marker for a number of epithelial malignancies including brain, prostate, colorectal, and lung [38, 39]. In HNSCC cell lines, CD133^hi^ cells display increased clonogenicity, tumor sphere formation and tumorigenicity in xenograft models when compared to their CD133^low^ counterparts [26, 40, 41]. While CD133 expression has been noted in primary human HNSCC tumors, quantitative xenotransplantation assays utilizing CD133+^hi^ cells from fresh tumors has yet to be performed. Given the artificial environment of cell culture, these findings will need to be substantiated using primary tumor samples before any definitive conclusions can be made about the usefulness of CD133 as a CSC marker in HNSCC.

#### 4.1.2. CD44

One of the most well-recognized CSC markers, is a large cell surface glycoprotein that is involved in cell adhesion and migration. It is a known receptor for hyaluronic acid and interacts with other ligands such as matrix metalloproteases [42, 43]. Initially identified as a solid malignancy CSC marker in breast cancer [18], Prince et al. demonstrated that CD44 expression could also be used to isolate a tumor subpopulation with increased tumorigenicity in HNSCC [23]. In their study they were able to show that as few as 5,000 CD44^hi^ HNSCC cells could form a tumor when transplanted into the flank of immunocompromised mice, whereas higher concentrations of CD44^low^ cells failed to form tumors. Additionally, these tumors recapitulated the original tumor's cellular heterogeneity and could be serially passaged, characteristics that define CSCs. 

Although CD44 expression enriches for cells with CSC properties, the relatively high number of cells required for tumor formation as compared with known CSC populations from other epithelial malignancies raises questions about whether CD44 expression alone is sufficient for isolation of a pure CSC population. For instance, in breast cancer, as few as 100 CSCs injected into the mammary fat pads of immunocompromised mice generated tumors [18]. It is important to note that in the Prince study, two-thirds of HNSCC samples were initially passaged through immunocompromised mice to generate a sufficient number of tumor cells for cell sorting, which has the potential for altering native CSC expression patterns. Using primary human tumor samples as well as utilizing a more natural host microenvironment through an orthotopic xenograft model [44] might reduce the number of cells needed to generate tumors. However, it is likely that expression of multiple cell surface markers or the combination of marker expression with functional assays will be needed to further enrich the CSC population.

### 4.2. Aldehyde Dehydrogenase Activity

 Aldehyde dehydrogenase (ALDH) is an intracellular enzyme normally present in the liver. Its known functions include the conversion of retinol to retinoic acids and the oxidation of toxic aldehyde metabolites, like those formed during alcohol metabolism and with certain chemotherapeutics such as cyclophosphamide and cisplatin [45–47]. ALDH activity is known to enrich hematopoetic stem/progenitor cells [48] and more recently has been shown to enrich cells with increased stem-like properties in solid malignancies [49–52]. Chen et al. showed that ALDH activity correlated with disease staging in HNSCC and that higher enzymatic activity correlated with expression of epithelial-to-mesenchymal transition (EMT) genes as well as enriching cells with CSC properties [53]. In addition, ALDH activity appears to enrich for CSCs in HNSCC to a higher degree than that currently provided by cell sorting based on surface antigen expression. Clay et al. demonstrated that as few as 500 ALDH^hi^ cancer cells could give rise to new HNSCC tumors when transplanted into immunocompromised mice, tenfold fewer cells than isolation by CD44 positivity. Most of the ALDH^hi^ cells were also CD44^high^, suggesting that ALDH activity defines a subset of HNSCC CD44^high^ cells with increased tumorigenicity [54].

### 4.3. Side Population

 Hoechst 33342 is a fluorescent DNA-binding dye that preferentially binds to A-T rich regions. It is actively pumped out of cells by members of the ATP-binding cassette (ABC) transporter superfamily. Once stained with Hoechst dye, cells can be sorted by fluorescent-activated cell sorting (FACS) based upon the activity level of these multidrug transporters. Originally noted to enrich bone marrow for long-term hematopoetic stem cells [55], this method has also been used to identify cells within solid tumors with increased tumorigenicity [21, 56, 57]. Side population (SP) cells from oral squamous cell carcinoma have been shown to have increased clonogenicity and tumorigenicity in xenotransplantation assays [25, 58]. Furthermore, HNSCC SP cells displayed higher expression of known stem cell related genes—Oct4, CK19, BMI-1 and CD44—and lower expression of involucrin and CK13, genes associated with a differentiated status [58].

### 4.4. Tumor Sphere Formation

Under serum-free culture conditions CSCs can be maintained in an undifferentiated state, and when driven toward proliferation by the addition of growth factors, form clonally derived aggregates of cells termed tumor spheres [22]. The ability of CSCs—but not the remaining tumor bulk—to form tumor spheres has been used extensively in neural tumors to identify populations enriched for CSCs. In HNSCC, these spheres have been shown to be enriched for stem markers, including CD44^hi^ [59], Oct-4, Nanog, Nestin, and CD133^hi^ [26, 60], as well as exhibiting increased tumorigenicity in orthotopic xenografts [60].

## 5. Cancer Stem Cells and Disease Progression

While there exists significant data defining the presence of CSCs within a variety of tumor types and many aspects of the cell and molecular biology of CSC have been elucidated, the manner in which this unique cell population influences clinical disease progression remains unclear. Given that metastases can be formed from implantation of a single tumor cell [61], it seems likely that CSCs, as the progenitor of all tumor cell types, would be responsible for metastatic spread. Central to the CSC hypothesis is the presence of a unique stem cell “niche” or environment necessary to support the growth of stem cells [62]. It has been shown that a premetastatic niche is established by the attraction of bone marrow derived cells to the future site of metastases by the secretion of factors from cancer cells and that blocking the creation of this premetastatic niche prevents metastases [63]. What these secreted factors are and whether they are secreted by CSCs or one of their progeny remains an open question; however, creation of this niche, possibly for the arrival of CSCs to form a metastasis, appears to be a crucial step in metastatic spread. 

The strongest evidence that CSCs are responsible for metastases comes not in HNSCC but in colorectal cancer. In this tumor, a unique CSC population that is CD26^hi^ appears to be tightly linked with metastases [64]. Not only are CD26^hi^ cells found in both primary and metastatic tumors, but the presence of CD26^hi^ cells in the primary tumor predicted future development of metastases. In a mouse xenograft study, CD26^hi^ CSCs implanted into the cecal wall of a nude mouse formed a tumor in the colon as well as liver metastases, while CD26^low^ CSCs formed a tumor at the site of implantation without developing liver metastases. Similarly, injection of CD26^hi^ CSCs into the portal vein led to liver metastases, while similar injection of CD26^low^ CSCs did not. Thus, these CD26^hi^ CSCs appear to be the cells responsible for metastatic spread in this tumor population.

Another stem cell marker, CD44, has also been implicated in metastatic spread and disease progression in HNSCC, although the CD44 story is more complex. Recently, three different isoforms, CD44 v3, v6, and v10, have been shown to be associated with progression and metastasis of HNSCC [65]. Increased CD44 v3 expression in primary tumors was associated with lymph node metastasis, while CD44 v10 expression was associated with distant metastasis and CD44 v6 expression was associated with perineural spread. In cell culture, blockade of these CD44 isoforms with isoform-specific antibodies inhibited cellular proliferation, with the greatest inhibition seen with blockade of CD44 v6. Finally, increased expression of CD44 v6 and v10 was associated with shortened disease-free survival. These studies suggest that alteration in CSC phenotype through variation in CD44 isoform expression may alter the interaction of CSCs with the surrounding microenvironment. This may allow CSCs to more readily invade surrounding tissues or metastasize, thereby promoting disease progression.

## 6. Cancer Stem Cells and Treatment Response

**Figure 3:**
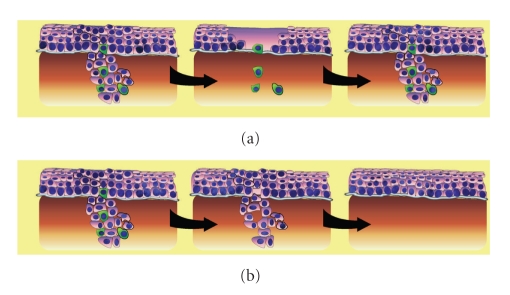
CSC Theory and Treatment Response. (a) Current chemotherapy and radiation treatment regimens with broad cytotoxic effects kill the bulk of differentiated tumor cells with preferential sparring of resistant CSCs, giving apparent volumetric reduction of tumor but subsequent recurrence. (b) Targeted therapy preferentially kills CSC leaving nonrenewing cells with eventual tumor death.

Aside from providing a model of disease progression and metastasis, CSCs have important implications regarding cancer treatment. While current chemotherapy and radiation treatment for HNSCC are focused on indiscriminate cytoreduction, the CSC hypothesis suggests that only by eliminating CSCs can cancer be treated effectively (Figure 3). However, there is substantial evidence that CSCs have inherent drug and radiation resistance, rendering most conventional therapies ineffective and explaining tumor recurrence despite significant reductions in tumor volume. By definition, stem cells must divide frequently and thus have highly stringent mechanisms to prevent and rapidly correct DNA damage. In the case of CSCs, there is evidence that these enhanced DNA protection and damage repair pathways lead to significant resistance to radiation and chemotherapy. 

The presence of a CSC population has thus far been implicated in radioresistance of multiple tumor types. For example, glioblastoma tumors that recur after radiation have been found to be enriched in CD133^hi^ CSCs [9]. Furthermore, this radioresistance of CD133^hi^ CSCs appears to be mediated through enhanced repair of DNA damage via the Chk1 and Chk2 kinases [9] and through enhanced cell longevity via the histone deacetylase SirT1 [11]. Similar results have been seen in a murine model of breast cancer, which showed that CSCs have substantially lower amounts of reactive oxygen species, leading to radioresistance [13]. HNSCC CSCs also demonstrated enhanced radioresistance in murine models that could be reduced by knockdown of the transcriptional repressor Bmi-1 [66]. However, the radioresistance of CSCs may not only depend on factors intrinsic to the CSC, but may also be driven by the unique CSC microenvironment. Cell cultures of CSCs from glioblastoma, pancreatic, breast, and colorectal carcinoma have been reported to have similar radiosensitivity to cell cultures that are not enriched for CSCs [8, 16].

In addition to radioresistance, CSCs also appear to mediate chemoresistance in multiple tumor types. There is evidence for chemoresistance of CSCs in lung [10], pancreatic [15], and breast carcinoma [12]. In HNSCC, CSCs were made more chemosensitive via knockdown of Bmi-1 [66]. Moreover, knockdown of CD44 increased the sensitivity of HNSCC cells to cisplatin, indicating that this CSC marker may be involved in meditating the response of these cells to chemotherapy. Other mechanisms of chemoresistance, such as drug efflux pumps, have been postulated but not yet identified in HNSCC. Future studies will be needed to further define resistance mechanisms in HNSCC CSCs to improve therapy and possibly prevent tumor spread or recurrence.

## 7. Prospectus

While the CSC theory is revolutionizing our understanding of tumor biology, many things remain to be elucidated regarding the role of CSCs in HNSCC. For starters, additional markers that enrich CSCs in other epithelial malignancies need to be evaluated in HNSCC. Only a limited number of CSC markers have been examined in HNSCC, and for each of these the reported number of marker-positive cells needed for tumor formation is significantly higher than what has been reported for other solid malignancies. It is clear that further purification of the CSC population in HNSCC is necessary. 

To date, only a few studies have used primary human HNSCC tissue for CSC marker identification [23, 54]. Prince et al. used primary tumor specimens in one-third of their samples, with the remaining two-thirds passaged initially through nude mice to generate a larger tissue sample prior to cell isolation [23]. Although this methodology has been used in other seminal studies for CSC marker identification [18] and was considered state of the-art at the time, increasing knowledge of the influence of the tumor microenvironment on the CSC clearly suggests avoidance of additional cell manipulation is preferred. In fact, in their follow-up study evaluating ALDH activity as a CSC marker in HNSCC all samples were from primary tumors [54]. Future studies should follow this example and concentrate on marker identification using primary tumor samples. Use of intermediate steps involving an artificial microenvironment has the ability to distort the naturally occurring CSC marker expression pattern. For similar reasons marker identification on HNSCC cell lines should be interpreted with caution. 

It is becoming more clear that the cellular heirarchy defined by the CSC theory is likely more complex than originally realized. Single marker identification may not be sufficient to identify a pure CSC population. In fact, as demonstrated in glioblastoma, CSCs can express or lack the traditional CSC marker CD133 yet still retain the functional characteristics that define a CSC [67]. It may be that expression of CSC markers evolves with disease progression and the accumulation of additional mutations or changes in response to therapy. It is possible that several distinct populations of CSCs with differing markers exist within a single tumor and that a combination of the clonal evolution and CSC models of tumorigenesis may be more appropriate than either alone to explain the behavior of some tumors. 

Additional work is also needed in defining the expression patterns of CSC markers in the endogenous setting. Little is known, especially in the head and neck, about the endogenous expression patterns of putative CSC markers. Further, it is unknown if the stem cell niche of head and neck mucosa is similar to that of cutaneous skin or if regional differences in CSC marker expression exist between subsites of the upper aerodigestive tract. Clearly an understanding of normal head and neck mucosal CSC marker expression is critical if attempts are to be made to selectively target CSCs as part of a treatment regimen for HNSCC. 

To date, most studies have focused on the identification of tumor cell populations enriched for cells with stem-like properties. We have little understanding of the significance of the markers used to identify these cells—whether they are simply markers of convenience or whether they have functional significance remains unknown. Examining the role these molecules may play in the tumorigenic, metastatic, and treatment resistance properties of CSCs is certainly a logical step on our way to discovering the mechanisms by which CSCs differ from the remaining tumor cell population.

The CSC theory offers an intriguing insight into why currently available therapies for head and neck cancer so often fail. While increasing evidence suggests that CSCs display increased resistance to multiple treatment paradigms inclusive of chemotherapy and radiation, the exact mechanisms by which they do this are incompletely understood. Focused research on mechanisms of treatment resistance in CSCs and whether they can be overcome will prove equally important as efforts to improve CSC identification.

Increasing our knowledge of the differences between CSCs, their differentiated progeny and normal endogenous stem cells, will translate into our ability to understand the seemingly complex cellular hierarchy in tumors. Ultimately an understanding of CSCs has the potential to identify novel targets for therapy and impact patient care.
